# Senescent mesenchymal stem/stromal cells in pre-metastatic bone marrow of untreated advanced breast cancer patients

**DOI:** 10.32604/or.2023.028104

**Published:** 2023-05-24

**Authors:** FRANCISCO RAÚL BORZONE, MARÍA BELÉN GIORELLO, LEANDRO MARCELO MARTINEZ, MARÍA CECILIA SANMARTIN, LEONARDO FELDMAN, FEDERICO DIMASE, EMILIO BATAGELJ, GUSTAVO YANNARELLI, NORMA ALEJANDRA CHASSEING

**Affiliations:** 1 Laboratorio de Inmunohematología, Instituto de Biología y Medicina Experimental (IBYME), Consejo Nacional de Investigaciones Científicas y Técnicas (CONICET), Buenos Aires, Argentina; 2 Division of Hematology and Medical Oncology, Department of Medicine, Weill Cornell Medical College, New York, USA; 3 Laboratorio de Regulación Génica y Células Madre, Instituto de Medicina Traslacional, Trasplante y Bioingeniería (IMeTTyB), Universidad Favaloro-CONICET, Buenos Aires, Argentina; 4 Facultad de Ciencias de la Salud, Universidad Nacional del Centro de la Provincia de Buenos Aires (UNCPB), Tandil, Buenos Aires, Argentina; 5 Servicio de Hematología, Hospital Militar Central, Buenos Aires, Argentina; 6 Servicio de Oncología, Hospital Militar Central, Buenos Aires, Argentina

**Keywords:** Mesenchymal stem/stromal cells, Senescence, Breast cancer, Bone marrow, Pre-metastatic niche, Bone metastasis

## Abstract

Breast cancer is the predominant form of carcinoma among women worldwide, with 70% of advanced patients developing bone metastases, with a high mortality rate. In this sense, the bone marrow (BM) mesenchymal stem/stromal cells (MSCs) are critical for BM/bone homeostasis, and failures in their functionality, transform the BM into a pre-metastatic niche (PMN). We previously found that BM-MSCs from advanced breast cancer patients (BCPs, infiltrative ductal carcinoma, stage III-B) have an abnormal profile. This work aims to study some of the metabolic and molecular mechanisms underlying MSCs shift from a normal to an abnormal profile in this group of patients. A comparative analysis was undertaken, which included self-renewal capacity, morphology, proliferation capacity, cell cycle, reactive oxygen species (ROS) levels, and senescence-associated β‑galactosidase (SA‑β‑gal) staining of BM-derived MSCs isolated from 14 BCPs and 9 healthy volunteers (HVs). Additionally, the expression and activity of the telomerase subunit TERT, as well as telomere length, were measured. Expression levels of pluripotency, osteogenic, and osteoclastogenic genes (*OCT-4*, *SOX-2*, *M-CAM*, *RUNX-2*, *BMP-2*, *CCL-2*, *M-CSF*, and *IL-6*) were also determined. The results showed that MSCs from BCPs had reduced ,self-renewal and proliferation capacity. These cells also exhibited inhibited cell cycle progression and phenotypic changes, such as an enlarged and flattened appearance. Additionally, there was an increase in ROS and senescence levels and a decrease in the functional capacity of TERT to preserve telomere length. We also found an increase in pro-inflammatory/pro-osteoclastogenic gene expression and a decrease in pluripotency gene expression. We conclude that these changes could be responsible for the abnormal functional profile that MSCs show in this group of patients.

## Introduction

Breast cancer is the most frequent carcinoma among women, constituting a quarter of all female cancer cases and causing over 2 million new cases worldwide in 2018 [1]. Unfortunately, despite advances in early detection, 20%–30% of BCPs in early clinical stage will experience relapse and die due to the complications arising from the dissemination of breast cancer cells from the primary tumor to the metastatic site [2,3]. Accumulating data suggest that 60%–70% of advanced BCPs develop osteolytic bone metastasis, with median overall survival of 3.4 years, a higher mortality rate, and several clinical complications, like hypercalcemia, pain, spinal cord compression, fracture, and reduced mobility that greatly impact on the quality of life [4–8]. Like other types of metastasis, the development of bone metastasis is an inefficient and complex process involving multiplesteps. The last step includes the BM/bone extravasation of disseminated breast cancer cells, the survival of a select few of them in a quiescent- or dormant-state for several years, and their later reactivation and development into micro/macrometastasis [9].

In 1889 Sir Stephen Paget proposed the “Seed and Soil” theory [10], making the first reference to the known PMN concept. This author postulated that tumor cells (seeds) can only grow in a supportive microenvironment (soil). A primary tumor is responsible for inducing this specialized environment distantly. Its main features include angiogenesis and vascular permeability, extracellular matrix remodelling, metabolic reprogramming of stromal cells, as well as an overproduction and release of both inflammatory and immunosuppressive cytokines and chemokines such as transforming growth factor-beta (TGF-β), vascular endothelial growth factor (VEGF), interleukin-6 (IL-6), IL-10, chemokine (C-C motif) ligand 2 (CCL-2), and granulocyte-macrophage colony-stimulating factor (GM-CSF), among others [11–13]. The establishment of this “fertile soil” for cancer cells, or PMN formation, is an essential previous step for the development of metastasis.

In particular, the BM consists of different compartments (niches), that are intimately connected. Therefore, each compartment must retain its functionality to maintain global BM/bone homeostasis. The MSCs are the key cellular components of these compartments, by regulating hematopoietic development and bone deposition and resorption. These cells, together with osteocytes and osteoblasts, are the primary source of nuclear factor κB receptor activating ligand (RANKL). On the surface of preosteoclasts, this ligand binds to its receptor, RANK, and stimulates osteoclast differentiation and activation, as well as bone resorption [14,15]. MSCs can also differentiate into osteoblasts, with several factors regulating this process, such as parathyroid hormone, RUNX family 2 (RUNX-2) transcription factor, osterix transcription factor, and the Wnt pathway [16,17]. Therefore, an adequate number of functional pool of MSCs in the BM/bone is required to prevent PMN formation.

MSCs are a diverse group of stromal cells that exhibit varying degrees of plasticity and proliferative potential. Despite the absence of a unique marker for identifying MSCs, the International Society for Cellular Therapy proposed some minimum criteria to define human MSCs. These include the capacity of adherence to plastic and develop fibroblastic colony-forming units (CFU-F) under standard culture conditions, the expression of >95% levels of CD105, CD73, and CD90 surface antigens and less than <2% of CD34, CD45, CD14 or CD11b, CD79a or CD19, and human leukocyte antigen (HLA)-DR surface molecules, as well as the ability to differentiate into chondroblasts, adipocytes and osteoblasts *in vitro* [18].

We previously found that MSCs obtained from BM of untreated advanced BCPs (stage III-B, without BM/bone metastasis) exhibited lower cloning efficiency to form CFU-F in primary cultures. Also, these MSCs developed smaller colonies with a low number of cells/CFU-F, with most of those cells lacking the spindle shape typically observed in stromal cells of HVs CFU-F. Additionally, these deficient colonies tended to be less confluent than the larger CFU-F from HVs [19–22]. Moreover, BCPs-MSCs presented lower differentiation capacity to osteocytes and adipocytes [21]. Furthermore, MSCs from these patients presented a highly expression of pro-osteoclastogenic factors like, matrix metalloproteinase 9 (MMP-9), CCL-2, and RANKL [23]. All the mentioned changes resemble those observed in MSCs affected by aging and senescence processes. In particular, MSCs in a senescent state, in response to DNA damage, trigger cell cycle arrest and have been associated to aging and age-related diseases, including cancer [24]. Senescence triggers a decrease of cell stemness, proliferation, and differentiation capabilities, and increase in cellular size and flattening, increase activity of SA-β-gal, inability to synthesize DNA, as well as several changes in gene and protein expression patterns [25,26]. Senescence is initiated at the molecular level by the retinoblastoma protein (Rb) and/or p53 pathways, which activate p16 and p21 cyclin-dependent kinase inhibitors [27,28]. Moreover, senescence negatively affects the secretome of MSCs, modifying their immunomodulatory properties, impeding MSC niche-supporting functions consistent with inflammaging development [28–30].

In accordance, previous works underlined the capacity of senescent human MSCs to promote either the proliferation or migration of breast cancer cells [31].

MSCs can become senescent under a range of stressors, including oxidative stress, heat shock, chemotherapeutic agents, and ionizing radiation [24]. Reactive oxygen species-induced premature senescence in MSCs is strictly related to telomer length. Telomeric DNA is synthesized by a specific multi-protein complex with reverse transcriptase activity, called telomerase [32]. When telomerase expression is low or absent, progressive telomeres shortening occurs in every cell division until the telomeres reach a critical length. At this point, the activation of the DNA damage checkpoint triggers senescence, thus preventing cells from further cycling [26,33]. In addition, telomerase must be located in the nucleus to fulfill its canonical function of telomere extension. In this sense, several authors demonstrated that oxidative stress leads to a decrease in the levels of human telomerase reverse transcriptase (hTERT) from the nucleus to the cytosol, via export through the nuclear pores [34–36]. We previously found a significant increase in lipid oxidation assessed by 2-thiobarbituric acid-reactive substances (TBARS) and ROS production in BM plasma and peripheral blood plasma from untreated BCPs [22].

Although the changes that occur in the BM/bone PMN are still being elucidated, little is known about the mechanisms that modify the MSCs, which have a critical role in establishing and regulating the PMN in BCPs. This work aims to study some of the metabolic and molecular mechanisms underlying MSCs shift from a normal to an abnormal profile in this group of patients.

## Materials and Methods

### Patients

All individuals consented to participate in this study, which was performed in accordance with the principles of the Helsinki Declaration. IBYME-Ethical Committee approved this investigation and informed consent.

Eligibility requirements included women 50 to 65 years, with infiltrative ductal breast carcinoma, clinical stage III-B (in accordance with the International Union against Cancer (UICC) TNM staging system) without previous primary tumor surgery, treatment free (no chemo-hormonal-, immuno- or radio therapy treatments) and menopausal. Exclusion criteria included BM or bone metastasis (bone metastasis were assessed by X-ray procedures and bone scintigraphy), osteoporosis, metabolic bone disease, such as vitamin D deficiency, thyroid disease, parathyroid disease, and kidney damage. The control cohort consisted of healthy volunteers (HVs) women 45 to 65 years, donors for allogeneic BM transplantation and were matched for menopausal status with patients. The study incorporated a total of 14 BCPs and 9 HVs.

### Isolation and preparation of BM-MSCs

BM samples were collected under local anesthesia from the posterior iliac crest into heparinized saline without preservatives (25 units/ml, Gibco, Grand Island, NY, USA). Histopaque (density = 1,075 gr/cm^3^, Sigma, St. Louis, MO, USA) density gradient was used to isolate mononuclear cells (MNCs) from BM samples. After centrifugation for 25 min at 340 g, BM light-density MNCs were harvested from the interface, washed twice in phosphate-buffered saline (PBS), and resuspended in α-minimal essential medium (α-MEM, cat. 11900024, Gibco, Grand Island, NY, USA) containing 2 mM L-glutamine (cat. 25030081, Gibco, Grand Island, NY, USA), 100 IU/ml antibiotic-antimycotic (cat. 15240062, Gibco, Grand Island, NY, USA) and 20% heat-inactivated fetal bovine serum (FBS) (cat. 16000044, Gibco, Grand Island, NY, USA) [supplemented α-MEM]. The MNCs suspension was counted in a 3% acetic acid solution and cell viability was determined by 0.04% trypan blue dye exclusion. Viable MNCs (10 × 10^6^) from BM of HVs and BCPs were plated in 25 cm^2^ tissue culture flasks with 10 ml of supplemented α-MEM. Cells were incubated at 37°C, 5% CO_2,_ and humidified environment. After 24 h, non-adherent cells were removed and the medium was renewed. Primary cultures were incubated until they reached 80% sub-confluence, renewing the medium every 7 days. Stromal cells from one flask of this first subculture were washed twice with PBS, harvested with a solution of trypsin-EDTA (0.05%–0.02% in PBS, respectively, cat. 15400054, Gibco, Grand Island, NY, USA) and re-plated in two 25 cm^2^ tissue culture flasks to proliferate until again reaching 80% confluence. After the second subculture, stromal cells were plated at low density (240 cells/cm^2^) in 25 cm^2^ flasks and incubated in supplemented α-medium for 12 days, the medium was renewed on day 6. Low cell density favours the growth of multipotential MSCs. Cells from the third subculture were isolated, and counted in a 3% acetic acid solution. Viability was determined with trypan blue. Surface phenotype analysis, triple lineage plasticity, and plastic adherence were performed to confirm that these cells exert the minimal criteria used to define MSCs [18].

### Study of BM infiltration with neoplastic cells

The absence of BM infiltration with neoplastic cells was confirmed by an immunocytochemistry system (cat. K0673, Dako, Carpinteria, CA, USA) and cell morphology analysis was performed by the Pappenheim technique. Aspirates from BM were stained with antibodies (Abs) against epithelial membrane antigen (cat. M0613, Dako, Carpinteria, CA, USA) and cytokeratins AE1–AE3 (cat. IR053, Dako, Carpinteria, CA, USA). Samples were considered positive for metastasis only if cells expressed EMA and cytokeratins AE1–AE3 and if cells were morphologically malignant. Isotype controls (cat. X0943, Dako, Carpinteria, CA, USA, and cat. 08-6599, ZYMED Laboratories, South San Francisco, CA, USA) were run in parallel using the same concentration of each Ab tested [37]. Experiments were repeated two times for each sample.

### Phenotypic characterization of MSCs from the third subculture

MSCs were suspended in PBS containing 1% bovine serum albumin (BSA, A7030, Sigma, Saint Louis, MO, USA), and labeled with the primary Ab against the following human antigens: CD105 (cat. FAB10971V, R&D Systems Inc., Minneapolis, MN, USA), CD90 (cat. FAB2067G, R&D Systems Inc., Minneapolis, MN, USA), CD73 (cat. FAB5795A, R&D Systems Inc., Minneapolis, MN, USA), CD79a (cat. FAB69201G, R&D Systems Inc., Minneapolis, MN, USA), CD11b (cat. FAB1699V, R&D Systems Inc., Minneapolis, MN, USA), and CD34 (cat. FAB7227P, R&D Systems Inc., Minneapolis, MN, USA). Isotype controls (cat. IC002V, cat. IC003G, cat. IC0041A, cat. IC002G, cat. IC002V, cat. IC002P, R&D Systems Inc., Minneapolis, MN, USA) were run in parallel using the same concentration of each Ab tested. The samples were incubated for 30 min at room temperature. At least 10,000 events were analyzed and compared with isotype controls by flow cytometry (FC; FACScanto II, BD Biosciences). FlowJo (v. X) software was used to create the histograms and density plots. Results were expressed as a percentage. Experiments were performed in duplicate with different MSC preparations.

### Differentiation of MSCs from the third subculture into osteoblasts, chondroblasts, and adipocytes

For MSCs plasticity studies, we followed the protocols previously described by our group [21,38]. Experiments were performed in duplicate for each sample.

### CFU-F assay from MSCs of the third subculture

The CFU-F assay was performed according to Chasseing et al. with modifications [21]. Viable MSCs from the third subculture (50 cells/cm^2^) of HVs and BCPs were placed in 25 cm^2^ tissue culture flasks containing 10 ml of supplemented α-medium. Cells were incubated at 37°C, 5% CO_2,_ in a humidified environment for 7 days. After this period, the medium was renewed. After another 7 days, the stromal cells were washed twice with PBS, fixed with 100% methanol (Merck, Darmstadt, Germany) for 15 min, and stained with pure Giemsa (cat. 48900 Sigma, Biopure, St. Louis, MA, USA) for 5 min at room temperature. Colonies with more than 50 cells were scored as CFU-F under a light microscope. The frequency of CFU-F is indicated by colony forming efficiency (CFE) defined as the number of CFU-F obtained every 1,250 MSCs seeded. For CFE evaluation, two flasks of each sample were analyzed. The number of MSCs per optical field of CFU-F (stromal cell density, SCD) was determined. For this purpose, ten pictures of different optical fields of each CFU-F culture were taken and analyzed with FIJI Software [39]. Morphological changes in CFU-F MSC cultures were also evaluated. Analysis of area, ellipse longitudinal, and horizontal axis values were carried out using three pictures obtained from three typical regions (three optical fields, 200X) of each CFU-F culture, evaluating 10 cells per photo and analyzed by FIJI Software [19].

### Mesenchymal stem/stromal cells from fourth subculture

Viable cells (3 × 10^3^/cm^2^) obtained from de third subculture of HVs and BCPs were plated in 25 cm^2^ tissue culture flasks with 10 ml of supplemented α-MEM. Cells were incubated at 37°C, 5% CO_2,_ in a humidified environment. Medium was renewed every 7 days until 80% confluence was reached. Then the cells were washed twice with PBS and harvested with a solution of trypsin-EDTA in PBS for further use.

### Proliferation assay

Viable cells of fourth subculture were seeded (5 × 10^3^ cells/well) and cultured in 96-well plates (cat. 4430100, Orange Scientific, Belgium) with 200 µl of supplemented α-MEM for 24 h. Then cultures were washed in PBS and incubated for 48 h in α-MEM without phenol red (α-MEM-PR, cat. 41061029, Gibco, Grand Island, NY, USA) plus 2 mM L-glutamine and 100 IU/ml antibiotic-antimycotic [supplemented α-MEM-PR]. Finally, the cells were washed in PBS and incubated over 48 h in supplemented α-MEM-PR with 5% FBS at 37°C, 5% CO_2,_ and humidity. Cell proliferation was evaluated by the CellTiter 96 AQueous Non-Radioactive Cell Proliferation Assay (cat. G5421, Promega, Madison, WI, USA) according to the manufacturer. Optical density (OD) was determined at 490 nm using a microplate reader. The value of each sample under study was obtained by subtracting the control OD (basal α-MEM condition culture) from its respective sample value. The differences between BCPs and HVs were evaluated. All experiments on the samples were carried out in triplicate.

### Cell cycle analysis

Viable cells from the fourth subculture were resuspended at 2 × 10^6^ cells/ml in PBS and fixed in ice-cold methanol for, at least, 24 h. Fixed cells were centrifugated at 340 g and each sample was resuspended in propidium iodide (PI) stain buffer, 200 mg of DNase-free RNase A (cat. GE101-01, Beijing TransGen Biotech Co., Ltd., Beijing, China), and 20 mg of PI (cat. 81845, Sigma Aldrich, Saint Louis, MO, USA), in PBS for 30 min. After staining, samples were analyzed using a FACScanto II (Becton Dickinson). Cell cycle analysis was determined using FlowJo (v. X). Experiments were repeated two times for each sample.

### Qualitative ROS staining

The levels of ROS were detected using fluorescent dyes MitoSOX^™^ Red (cat. M36008, Molecular Probes, Invitrogen^™^, Eugene, OR, USA) and CellROX® Oxidative Stress Reagents (cat. C10444, Thermo Fisher, Carlsbad, CA, USA). Fluorescent intensity was performed by incubating viable cells of fourth subculture at 37°C for 20–30 min following the manufacturer’s protocols and directly analyzed by flow cytometry (FACScanto II; Becton Dickinson). Fluorescence mean intensity on viable cells were selected (DAPI negative, cat. D9542, Sigma Aldrich, Saint Louis, MO, USA) in the chosen fluorescence channel (PE or FITC) and determined using FlowJo (v. X, Tree Star, Inc. Ashland, OR, USA). Experiments were repeated two times for each sample.

### SA-β-gal staining

Viable cells of fourth subculture were seeded (1 × 10^4^ cells/well) and cultured in 24-well plates (cat. 4430300, Orange Scientific, Belgium). After 24 h, the cultures were washed in PBS and incubated for 72 h more in supplemented α-MEM. Finally, cells were washed in PBS, fixed in 3% formaldehyde, and washed again in PBS and incubated for 16 h at 37°C without CO_2_ with fresh SA-β-gal staining solution: 1 mg/mL 5-bromo-4-chloro-3-indolyl b-D-galactopyranoside (X-Gal) (cat. 11680293001, Sigma Aldrich, Saint Louis, MO, USA), 150 mM NaCl, 2 mM MgCl_2_, 40 mM citric acid/Na phosphate buffer (pH 6), 5 mM potassium ferrocyanide, and 5 mM potassium ferricyanide. The stained cells were viewed and photographed with an inverted Olympus CKX41 (Olympus, Shinjuku-ku, Tokyo, Japan) microscope with 20X/0.4 objective using Olympus camera Q-Color 5 [40]. Experiments were repeated two times for each sample.

### Quantitative telomerase repeat amplification protocol assay and telomere length assay

TERT activity was assessed in cell protein extracts of fourth subculture using a real-time polymerase chain reaction (q-PCR) assay that measures the ability of telomerase to extend an exogenous primer (TS and ACX, Table 1), as previously described by Yannarelli et al. [41,42]. Results are expressed as percentage relative to MCF-7 (ATCC® HTB-22^™^) activity. A well-known breast cancer cell line with positive expression and high activity of TERT.

**Table 1:** DNA primers sequences

Primer	Sequence (5′-3′)	Size (bp)
ACX	GCGCGGCTTACCCTTACCCTTACCCTAACC	—
TS	AATCCGTCGAGCAGAGTT	
TELO-FWR	CGGTTTGTTTGGGTTTGGGTTTGGGTTTGGGTTTGGGTT	—
TELO-REV	GGCTTGCCTTACCCTTACCCTTACCCTTACCCTTACCCT	
GAPDH-forward	CCACATCGCTCAGACACCAT	178
GAPDH-reverse	CATGGGTGGAATCATATTGGA	
NANOG-forward	TGAACCTCAGCTACAAACAG	154
NANOG-reverse	TGGTGGTAGGAAGAGTAAAG	
SOX-2-forward	AGCTACAGCATGATGCAGGA	126
SOX-2-reverse	GGTCATGGAGTTGTACTGCA	
OCT-4-forward	AGCGAACCAGTATCGAGAAC	139
OCT-4-reverse	TTACAGAACCACACTCGGAC	
TERT-forward	GCCTGAGCTGTACTTTGTC	106
TERT-reverse	CGTGTTCTGGGGTTTGATG	
M-CAM-forward	TGAGGAGGTCGCTACCTGTGT	129
M-CAM-reverse	GACTCCACAGTCTGGGACGA	
BMP-2-forward	TCCATGTGGACGCTCTTTCA	75
BMP-2-reverse	GGTCGACCTTTAGGAGACCG	
RUNX-2-forward	CACAAGTGCGGTGCAAACTT	82
RUNX-2-reverse	GGTAGTGACCTGCGGAGATT	
CCL-2-forward	GAAAGTCTCTGCCGCCCTT	88
CCL-2-reverse	GGCATTGATTGCATCTGGCTG	
M-CSF-forward	GATTCTCCCTTGGAGCAACCA	93
M-CSF-reverse	GCGTCCAGCTTAGAATTCCCT	
IL-6-forward	TTCCAAAGATGTAGCCGCCC	92
IL-6-reverse	CTGAGATGCCGTCGAGGATG	

Relative telomere length was measured from genomic DNA samples by a q-PCR method using specific primers (TELO-FWR and TELO-REV, Table 1), as described by O’Callaghan et al. [43]. DNA isolated from MCF-7 cells was used as a reference control. The PCR data were analyzed with the comparative cycle threshold (Ct) method (2^−ΔΔCt^). Briefly, after calibration, the Ct of the telomeric sequence was subtracted by the Ct of the reference gene to calculate ΔCt. The ΔCt of the sample was subtracted from the ΔCt of the reference control to calculate the ΔΔCt. Finally, the relative telomere length was calculated using the 2^−ΔΔCt^ equation. This method measures the relative expression of the telomeric sequence in comparison to a reference gene. Experiments were repeated two times for each sample.

### Western blotting

Western Blotting (WB) was performed to evaluate protein expression levels in cell lysates. Briefly, viable cells from the fourth subculture (1 × 10^5^) were resuspended in 100 µl of Thermo Scientific^™^ NP-40 lysis buffer (cat. J60766.AP, Thermo Scientific^™^, Waltham, MA, USA) containing 50 mM Tris-HCl (pH 7.4), 150 mM NaCl, 1% NP-40, 5 mM EDTA and 1X complete^™^ Protease Inhibitor Cocktail (cat. 11873580001, Roche, Mannheim, Germany). Lysates were incubated on ice for 30 min and centrifuged at 10,000 g for 20 min at 4°C. Supernatant was collected. Protein concentrations were determined using DC Protein Assay (cat. 5000111, Bio-Rad, Hercules, CA, USA). Proteins (50 µg) were resolved in sodium dodecyl sulfate-polyacrylamide (SDS-PAGE) 12% gel. The resolved protein bands were electroblotted onto a polyvinylidene fluoride membrane (PVDF, cat. 1620177, Bio-Rad, Hercules, CA, USA), and their molecular weights were determined using the PageRuler^™^ (cat. 26616, Thermo Scientific^™^, Vilnius, LT, Lithuania), and blocked using 1XTris-buffered saline (TBS) with 0.1% TWEEN 20 (T-20) and 5% (w/v) non-fat dried milk for 45 min and hybridized overnight at 4°C with primary Ab against hTERT (monoclonal mouse IgG1, dilution 1:125, cat. MAB6595, R&D Systems, MN, USA). After washing with TBS 0.01% T-20 buffer, membranes were incubated with horseradish peroxidase (HRP)-conjugated with anti­mouse IgG secondary Ab (polyclonal goat IgG, dilution 1:1000, cat. HAF007, R&D Systems, MN, USA) and washed five times for 5 min with TBS 0.01 % T-20 buffer. Bound Abs were visualized using Amersham ECL Prime Western Blotting Detection Reagent system (cat. RPN2232, GE Healthcare, Marlborough, MA, USA), according to the manufacturer’s instructions. Bands were quantified by densitometry using FIJI software. Actin (polyclonal rabbit IgG, dilution 1:1,000; cat. sc-1616; Santa Cruz Biotechnology, Inc., Santa Cruz, CA, USA) levels were used as a loading control. Experiments were repeated two times for each sample.

### Quantitative RT-PCR

Total RNA was extracted from cells from the fourth subculture using TRI Reagent® (MCR) and 1 µg RNA was reverse transcribed into cDNA using random primers (High-Capacity cDNA Reverse Transcription Kit, Applied Biosystems, Foster City, CA, USA). Samples were assayed using FS UNIVERSAL SYBR GREEN MASTER ROX master mix (cat. 04913850001, ROCHE, Mannheim, Germany) on a CFX96^™^ TOUCH REAL-TIME PCR real-time PCR system (Bio-Rad, Hercules, CA, USA) under standard cycling conditions, followed by a melting curve analysis. The threshold cycle (Ct) values were normalized against the reference gene glyceraldehyde-3-phosphate dehydrogenase (GAPDH) and data are presented as the fold change in gene expression, relative to the HVs group. Primer sequences are provided in Table 1. Experiments were performed in duplicate for each sample.

### Statistical analysis

Results were expressed as the mean ± standard error (SE) when appropriate. Shapiro-Wilk test for normality was used to determine whether data were normally distributed and then analyzed with the pertinent test. For parametric data, differences between groups were tested by using an unpaired *t*-test with Welch’s correction.

For non-parametric data, differences between groups were analyzed by using a Mann–Whitney test. All statistical tests were two-tailed. Statistical analyses were performed by using GraphPad 6 Prism software (GraphPad Prism version 6.01, GraphPad Software, La Jolla CA, USA). Differences were considered statistically significant when *p* < 0.05.

## Result

### Study of BM infiltration with neoplastic cells

All BCPs tested negative for metastasis in routine diagnostic tests. Moreover, none of the BM aspirates showed any evidence of neoplastic cell infiltration. Furthermore, breast cancer cells were absent in all CFU-F cultures of MSCs from the third subculture.

### Phenotypic characterization and multilineages differentiation potential of MSCs

**FIGURE 1:**
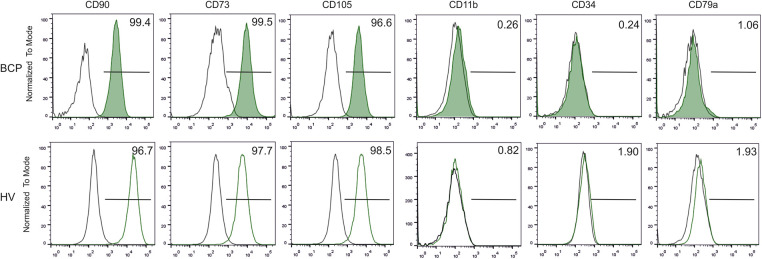
Phenotypic characterization of bone marrow-derived mesenchymal stem/stromal cells (BM-MSCs). Representative flow cytometry histograms of MSC surface antigens from a breast cancer patient (BCP) and healthy volunteer (HV). (□) Isotype control.

Phenotypic characterization of MSCs from the third subculture was performed using FC analysis of cell surface markers. Positive immunostaining (>95%) was consistently obtained for CD73, CD105, and CD90 without the expression of CD11b, CD34, and CD79a indicating their MSC nature (Fig. 1).

Cells from both groups were able to differentiate into osteoblasts, chondroblasts, and adipocytes (data not shown). Therefore, MSCs used in this study comply with the minimal criteria defining MSCs [18].

### Limited self-renewal and altered morphology of MSCs

**FIGURE 2:**
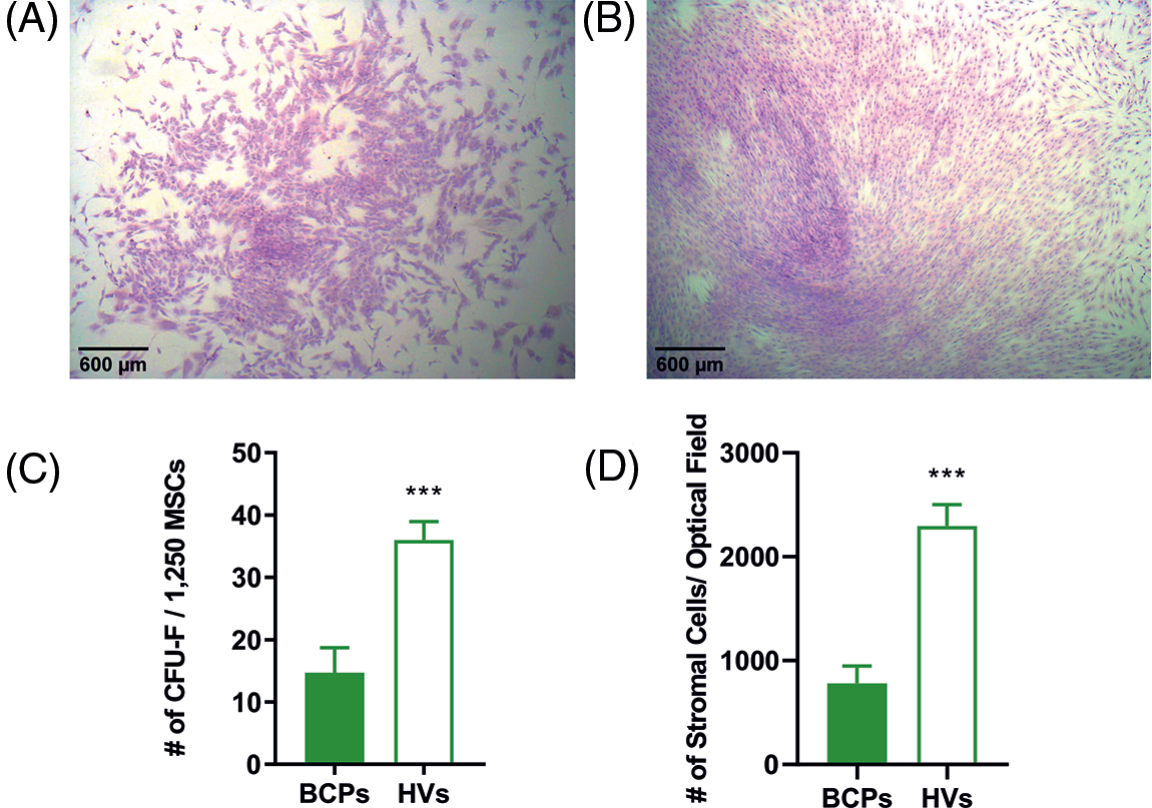
Limited self-renewal of mesenchymal stem/stromal (MSCs) from breast cancer patients (BCPs). (A) Colony forming units-fibroblastic (CFU-F) size observed for a BCP. (B) CFU-F size observed for a healthy volunteer (HV). Giemsa staining (40 X). (C) CFU-F assay: number of CFU-F observed in BCPs (*n *= 14) and HVs (*n *= 7). Values are expressed as mean ± SE. Statistical analysis: unpaired *t*-test with Welch’s correction. Asterisks indicate a significant difference (***p = 0.0004). (D) Number of stromal cells per microscope optical field (stromal cell density, SCD) in each CFU-F from BM of BCPs (*n *= 13) and HVs (*n *= 6). Statistical analysis: nonparametric Mann–Whitney test. Asterisks indicate a significant difference (***p = 0.0040).

Colony forming efficiency (CFE = # of CFU-F/ 1,250 MSCs) and CFU-F size (SCD) of MSCs from BCPs were lower than those of HVs (Fig. 2), in accordance with previous works that indicate a deficiency in the self-renewal of MSCs from BM primary cultures of cancer patients compared to HVs [19]. In agreement with our previous observations, the morphology of stromal cells may be used as a surrogate marker to predict individual stromal cell maturational arrest [19].

**FIGURE 3:**
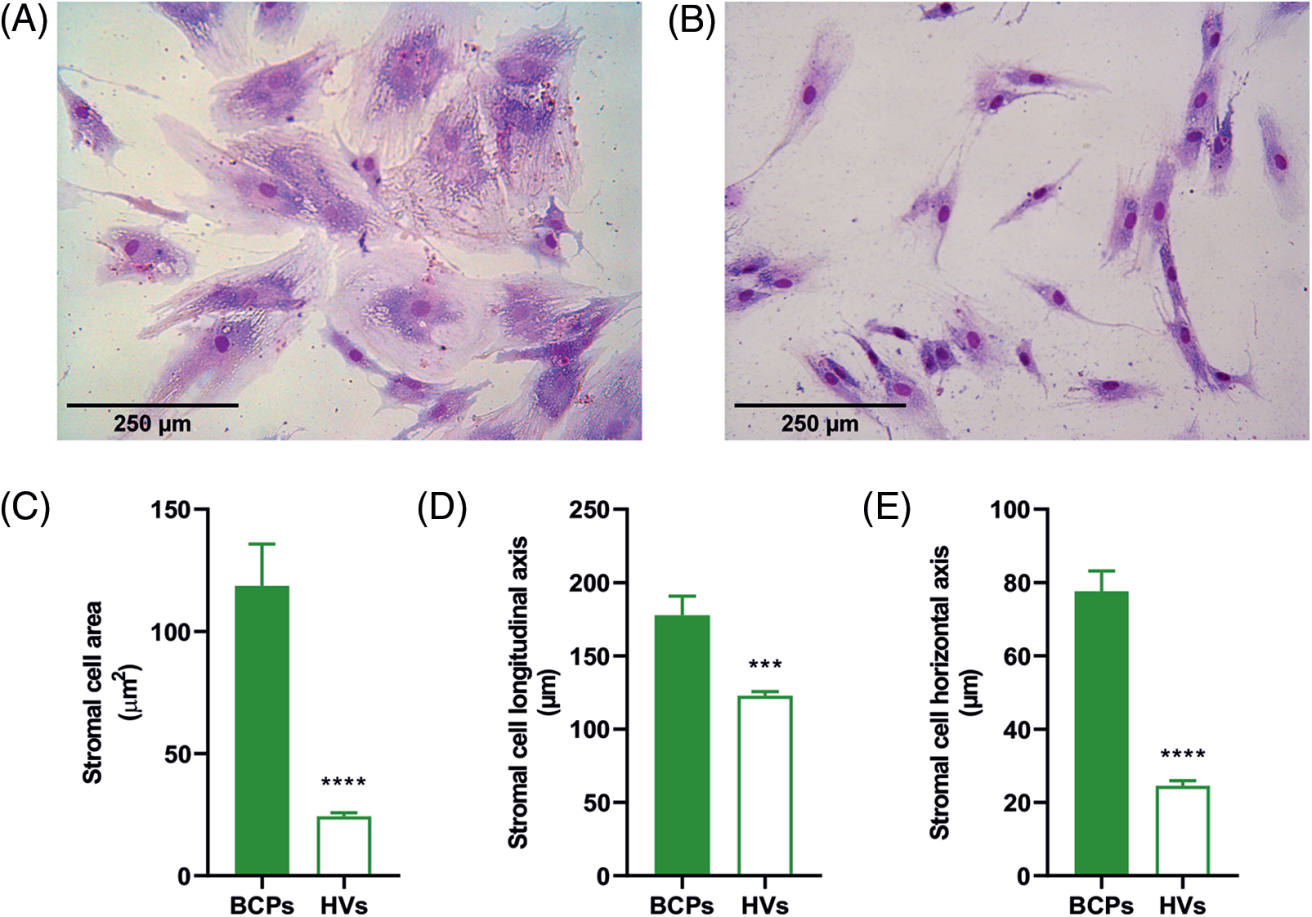
Altered morphology of mesenchymal stem/stromal (MSCs) from breast cancer patients (BCPs). (A) and (B) MSCs size and shape observed in colony forming units-fibroblastic (CFU-F) assay of a BCP and a healthy volunteer (HV). Giemsa staining (200×). (C) Area of stromal cells in typical regions of each CFU-F culture from BCPs (*n* = 13) and HVs (*n* = 6). (D) Longitudinal axis of stromal cells in typical regions of CFU-F cultures from BCPs (*n* = 13) and HVs (*n* = 6). (E) Horizontal axis stromal cells in typical regions of CFU-F culture from BCPs (*n* = 13) and HVs (*n* = 6). The values are expressed as Mean ± SE. Statistical analysis: nonparametric Mann–Whitney test. Asterisks indicate a significant difference (****p < 0.0001 and ***p < 0.0003).

To estimate the size of individual stromal cells of the CFU-F, three different measures were utilized, which consisted of surface area, longitudinal axis, and horizontal axis (Fig. 3). These three markers consistently showed that CFU-F of BCPs contain cells with a large area, and both large horizontal and longitudinal axis, as well as an enlarged and flattened appearance, indicating a senescent phenotype (“blanket cell type morphology,” increased size cells), while stromal cells cultured from BM of HVs, exerted a uniform fibroblast-like shape (spindle shape morphology, small cells) (Figs. 3A and 3B).

### Inefficient proliferation capacity and inhibited cell cycle progression of MSCs from BCPs

**FIGURE 4:**
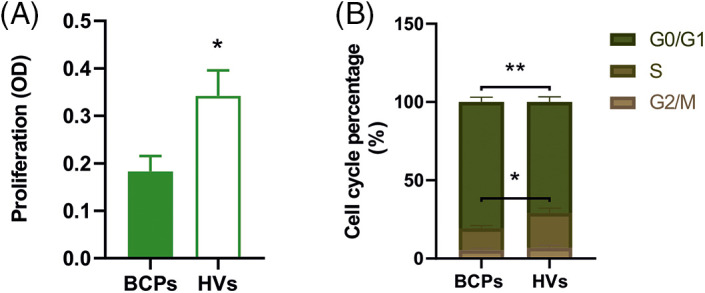
Inefficient proliferation capacity and inhibited cell cycle progression of mesenchymal stem/stromal (MSCs) from breast cancer patients (BCPs). (A) Proliferation of MSCs from BCPs (*n* = 10) and healthy volunteers (HVs, *n* = 9). Values are expressed as the mean ± SE. Statistical analysis: unpaired *t*-test with Welch’s correction. Asterisks indicate a significant difference (*p = 0.0240). (B) Cell cycle analysis. Percentage of MSCs in each phase of the cell cycle (G0/G1, G2/M, and S phase) from BCPs (*n* = 10) and HVs (*n* = 9). The values are expressed as Mean ± SE. Statistical analysis: unpaired *t*-test with Welch’s correction. Asterisks indicate a significant difference (*p = 0.0400 and **p = 0.0090).

A high proliferation rate is one of the main characteristics of MSCs. Cell growth rate over a 5-day period was significantly decreased in MSCs from BCPs when compared with HVs (Fig. 4A).

Regarding cell cycle analysis, the percentage of MSCs in G0/G1 and S phases from BCPs were different to MSCs from the HVs group. As shown in Fig. 4B, MSCs from BCPs displayed an extended G0/G1 phase and a shortened S phase.

### High ROS levels and a senescent phenotype of MSCs from BCPs

**FIGURE 5:**
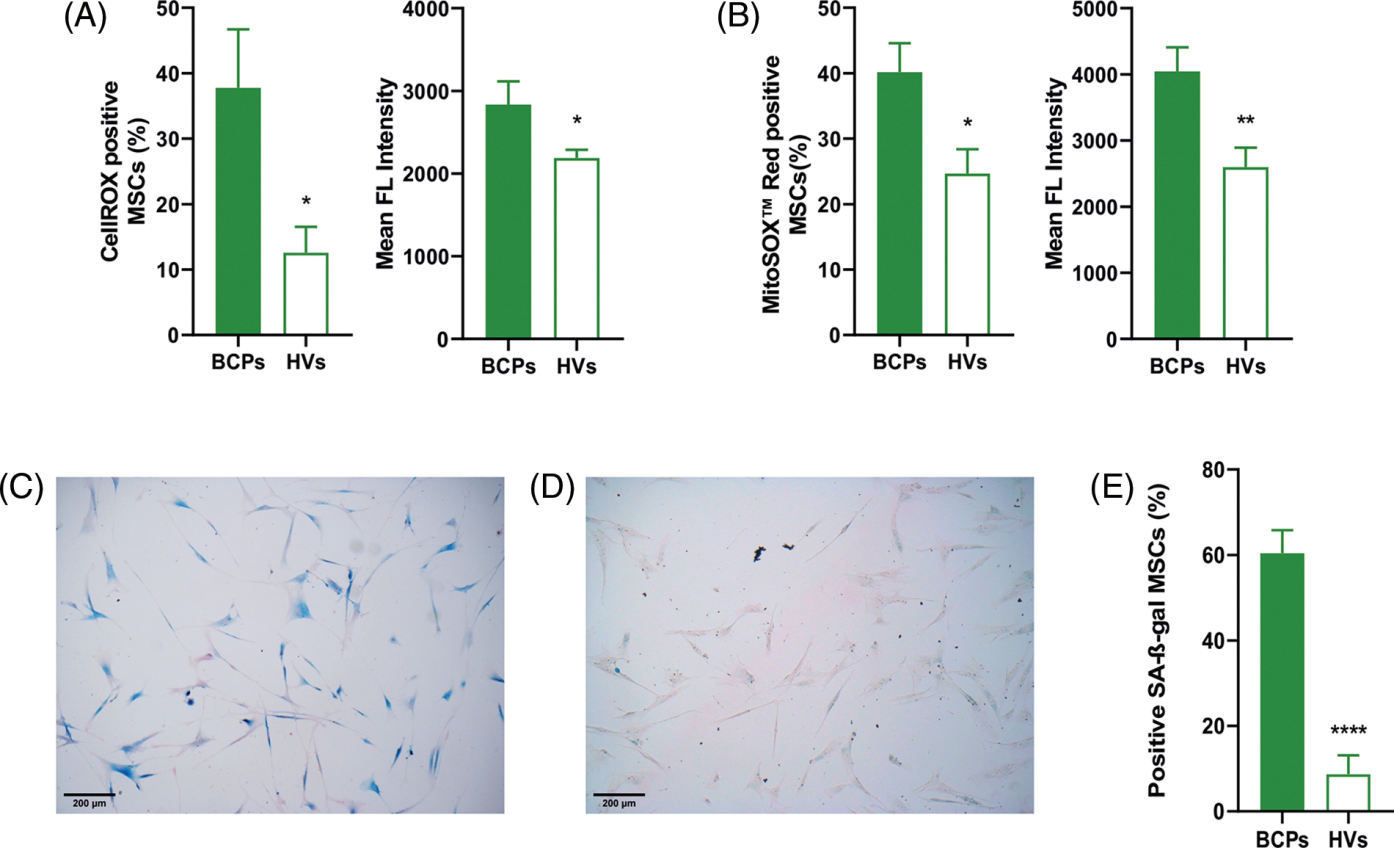
Mesenchymal stem/stromal cells (MSCs) from breast cancer patients (BCPs) present high ROS levels and senescent phenotype. (A) Cellular reactive oxygen species (ROS) levels. CellROX staining and mean fluorescent intensity of MSCs from BCPs (*n* = 6) and healthy volunteers (HVs, *n* = 8). The values are expressed as Mean ± SE. Statistical analysis: unpaired *t*-test with Welch’s correction. Asterisks indicate a significant difference (*p = 0.0365 and **p = 0.0442), respectively. (B) Mitochondrial ROS levels. MitoSox red staining and mean fluorescent intensity of MSCs from BCPs (*n* = 9) and HVs (*n* = 9). The values are expressed as Mean ± SE. Statistical analysis: unpaired *t*-test with Welch’s correction. Asterisks indicate a significant difference (*p = 0.0162 and **p = 0.0098, respectively). (C) Senescent associated‑β‑galactosidase (SA-β‑gal) staining assays. Representative images from a BCP. (D) SA‑β‑gal staining assays. Representative images from a HV (100 X). (E) Presence of senescent MSCs from BCPs (*n* = 9) and HVs (*n* = 6). The values are expressed as Mean ± SE. Statistical analysis: unpaired *t*-test with Welch’s correction. Asterisks indicate a significant difference (****p < 0.0001).

Our previous reports had showed that untreated advanced BCPs presented elevated levels of TBARS in BM and peripheral blood plasma, indicating an increase in both lipid oxidation and ROS production [22]. We measured the cellular and mitochondrial ROS levels in MSCs. BCPs group showed a significantly higher percentage of positive cells for both CellROX® and MitoSOX^™^ probes (Figs. 5A and 5B). Additionally, we found an elevated Mean Fluorencent Intensity (level of ROS per cell) for both probes.

In this sense, a quantitative measurement of the mean intensity from the samples showed a markedly enhanced mitochondrial superoxide production in MSCs from BCPs (Figs. 5A and 5B). Moreover, these MSCs contained a significantly larger porcentage of SA-β‑gal positive cells, which indicates cell senescence (Figs. 5C and 5D).

### Altered TERT and telomere state of MSCs from BCPs

**FIGURE 6:**
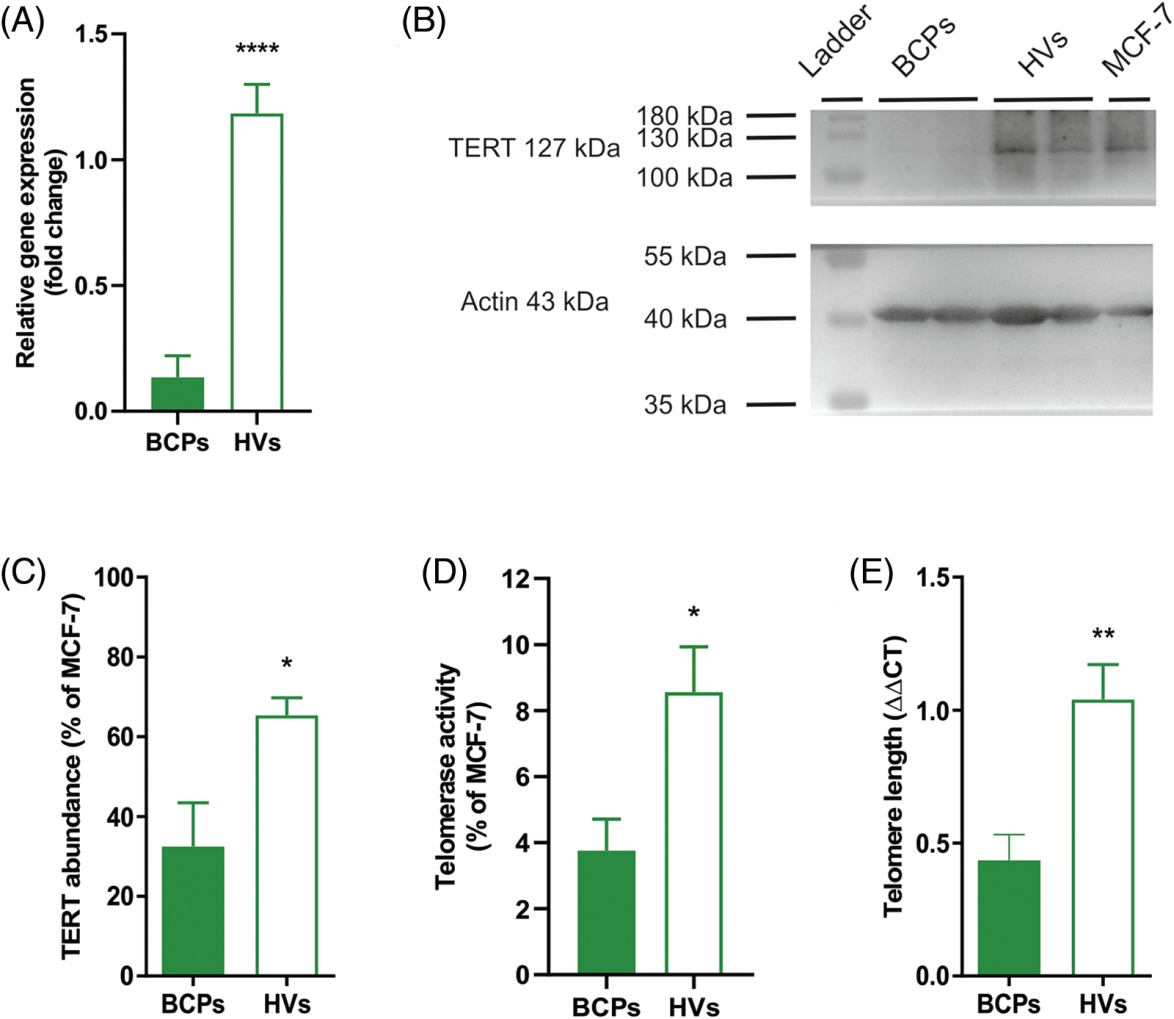
Mesenchymal stem/stromal cells (MSCs) from breast cancer patients (BCPs) exhibit low telomerase expression and activity and short telomeres. (A) Quantitative RT-PCR analysis of telomerase reverse transcriptase (TERT) gene expression in MSCs from BCPs (*n* = 6) and healthy volunteers (HVs) (*n* = 5). The values are expressed as Mean ± SE. Statistical analysis: unpaired *t*-test with Welch’s correction. Asterisks indicate a significant difference (****p < 0.0001). (B) Representative immunoblot from MSCs extracts from BCPs and HVs, using antibodies against TERT and actin. (C) Protein abundance of TERT from MSCs extracts from BCPs (*n* = 6) and HVs (*n* = 7). TERT and Actine expression were analyzed by immunoblot and images were analyzed by densitometry. The values are expressed as Mean ± SE. Statistical analysis: unpaired *t*-test with Welch’s correction. Asterisks indicate a significant difference (*p < 0.0200). (D) Relative amounts of telomerase activity assayed by Q-TRAP. Results are expressed as percentage relative to MCF-7 activity. BCPs (*n* = 11) and HVs (*n* = 8). The values are expressed as Mean ± SE. Statistical analysis: unpaired *t*-test with Welch’s correction. Asterisks indicate a significant difference (*p < 0.0130). (E) Telomere length measurement by real-time quantitative PCR. DNA isolated from MCF-7 (ATCC® HTB-22™) cells was used as a reference control. The PCR data were analyzed with the comparative cycle threshold (Ct) method (2^−ΔΔCt^). BCPs (*n* = 9) and HVs (*n* = 6). The values are expressed as Mean ± SE. Statistical analysis: unpaired *t*-test with Welch’s correction. Asterisks indicate a significant difference (**p < 0.0040).

We found significantly lower *TERT* mRNA in MSCs from BCPs when compared with HVs (Figs. 6A–6C). This correlated with lower TERT protein expression levels in BCPs-MSCs. Moreover, MSCs from BCPs showed decreased TERT activity and significantly shorter telomeres (2.4-fold difference) than MSCs from HVs (Fig. 6D and 6E).

### Altered gene expression of self-renewal, multipotency, osteogenic and osteoclastogenic factors in MSCs of BCPs

**FIGURE 7:**
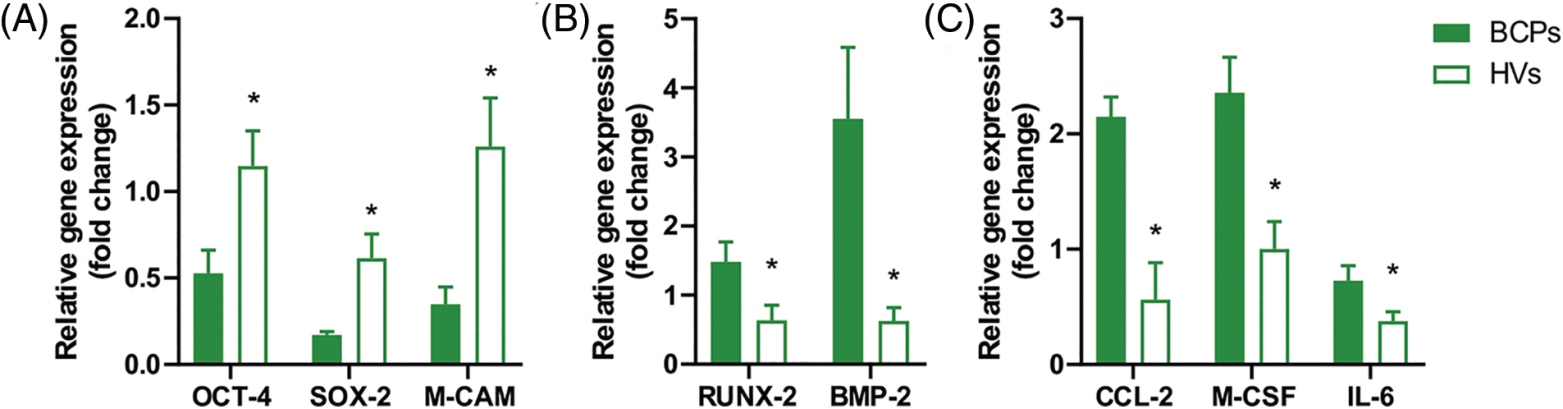
Expression of pluripotency, osteogenic and osteoclastogenic factors in mesenchymal stem/stromal cells (MSCs). (A) Gene expression of pluripotency factors in MSCs from breast cancer patients (BCPs) (*n* = 8) and healthy volunteers (HVs) (*n* = 7). Expression of *OCT-4*, *SOX-2*, *M-CAM* by quantitative real-time polymerase chain reaction (q-PCR). (B) Gene expression of osteogenic factors in MSCs from BCPs (*n* = 7) and HVs (*n* = 7). Expression of *RUNX-2* and *BMP-2* by quantitative real-time PCR. (C) Gene expression of osteoclastogenic factors (*CCL-2*, *M-CSF*, and *IL-6*) in MSCs from BCPs (*n* = 7) and HVs (*n* = 7). All the results were normalized against a set of reference genes and plotted relative to the expression level of MSCs from HVs. The values are expressed as Mean ± SE. Statistical analysis: unpaired *t*-test with Welch’s correction. Asterisks indicate a significant difference (*p < 0.0500).

The multipotent and self-renewal properties of MSCs had been previously related to the expression of pluripotency factors. In this sense, we found that the pluripotency factors *OCT4*, *SOX2*, and *M-CAM* (CD146) were downregulated in MSCs of BCPs when compared with HVs (Fig. 7A). Interestingly, the expression of the osteogenic factors *RUNX-2* and *BMP-2*, was upregulated (Fig. 7B). In addition, gene transcription of the osteoclastogenic factors *CCL-2*, *M-CSF* and *IL-6* was upregulated in MSCs of BCPs (Fig. 7C).

## Discussion

Advanced breast cancer frequently metastasizes to the bone. Both the bone and BM provide an exceptional soil for tumor growth, including those niches occupied by hematopoietic stem cells, MSCs, and osteoblasts. We had previously reported that BM-MSCs reservoir of untreated advanced BCPs may not be adequate to achieve bone regeneration and regulate the production and activity of mature osteoclasts and osteoblasts; and may be responsible for generating a PMN adequate for the extravasation, invasion, migration and proliferation of tumor cells in the BM and bone [19–21]. Changes in the peripheral blood and BM from untreated advanced BCPs were also correlated with the establishing of bone metastasis [37]. Identifying those mechanisms by which PMN contributes to tumor seeding and outgrowth is of key importance [37,44]. However, little is known about the molecular, metabolic, and phenotypical changes responsible for BCPs-MSCs modifications. Here we provide compelling evidence of those changes in dysfunctional MSCs of untreated advanced BCPs, without surgery and metastasis.

Low-density MNC cultures from BM are characterized by their plastic adherence and the formation of colonies of adherent fibroblast-like MSCs. Each colony arising from a single precursor cell, termed CFU-F, represents *in vitro* enumeration of a clonogenic subset of *in vivo* marrow MSCs populations. Kuznetsov et al. described that CFU-F populations are not homogeneous but rather contain a hierarchy of progenitors, including multipotential MSCs and committed progenitors [45].

In addition, the authors described that in numerous pathologies, the formation of CFU-F colonies is significantly different from normal, which may provide valuable insights into the pathogenetic mechanisms underlying BM/bone diseases [45]. In this paper, we report differences in cloning efficiency and morphology of MSCs isolated from BCPs when compared with HVs, in accordance with previous reports [19,21]. Moreover, we observed changes in the morphology of these cells, like enlarged and flattened appearance. It is known, that enlarged and irregularly shaped cells, resulting from cytoskeleton rearrangement, represent a hallmark of aging, associated with senescence [46,47]. Furthermore, the increase of SA-β-gal activity, inefficient proliferation capacity and inhibited cell cycle progression that we observed in MSCs from BCPs, are distinguishing marks of the senescent process [28,48,49]. Turinetto et al. described that senescent cells that typically exhibit SA-β-gal activity experience irreversible growth arrest while maintaining metabolic activity [28].

In this paper, we showed, that, not only phenotypic and functional differences existed between BCPs-MSCs and HVs in enriched-MSCs culture conditions, but that those differences were exacerbated when compared with previous results from BCPs-MSCs primary cultures [19].

These observations suggest that BCPs BM pool of MSCs experienced premature aging and senescence. Additionally, Liu et al. showed that aging is caused by the chronologic aging of the host, and is accelerated by conditions such as obesity and systemic inflammation [29].

This is relevant if we consider that some authors like Schäfer et al. referred to tumors as “wounds that never heal” due to their continuous release of inflammatory mediators into the milieu [50]. Our results confirm the ability of primary breast tumor to modify from distance the population of MSCs in the BM.

Furthermore, oxidative stress was reported to be involved in a broad spectrum of pathologies, including chronic inflammation and various types of cancers [51–53]. Different stressors can trigger the senescent program in MSCs, including oxidative stress, defined as the imbalance between ROS formation and the antioxidant defense in the body [28]. The ROS are produced mostly through the internal mitochondrial electron transport chain, which accounts for approximately 90% of their total production. An excess or accumulation of ROS can damage biomolecules, including DNA, protein, and lipids, thus causing MSCs dysfunction and triggering cellular senescence [29,54]. Moreover, senescent MSCs with elevated levels of ROS, cause persistent DNA damage response activation, that favours positive feedback loop in the progression of senescence [55]. In this study, we showed that MSCs from BCPs have increased levels of ROS both in the whole cell and mitochondrial compartment (which correspond to superoxide overproduction), when compared with HVs. It is known that aged and senescent cells have dysfunctional mitochondria with a disrupted electron transport chain, leading to modifications in the ratio of AMP/ATP as well as an increase in ROS production.

Furthermore, increased levels of ROS can induce nuclear DNA damage and damage mitochondria, giving rise to a positive feedback loop that reinforcing the cell cycle arrest [56]. Autophagy is critical for preserving cellular homeostasis under physiologic and pathologic conditions by removing damaged cellular components, allowing MSCs to avoid the transition from a reversible quiescent (G0/G1) state to irreversible senescent state [57,58]. Increased ROS reduces the autophagic capacity of MSCs which results in the loss of proteostasis and increases mitochondrial activity, metabolism, and oxidative stress [59], thus diminishing the regenerative potential and self-renewal capacity of MSCs [29].

The catalytic telomerase subunit TERT is closely related to aging, senescence, and oxidative stress processes. As previously reported [60], we found a decrease in TERT levels of expression and activity, and a consequent telomere shortening in MSCs from BCPs. In accordance, Lopez-Otín et al. showed that telomere attrition is one of the hallmarks of aging [61]. Moreover, Ahmed et al. showed that chronic oxidative stress disrupts telomere maintenance at two distinct levels. On the one hand, it increases the basal rate of telomere shortening. When telomeres reach a critically short length, they induce a DNA damage response that can transiently halt cell cycle progression through stabilizing p53 and transcriptional activation of the cyclin-dependent kinases (CDK) inhibitor p21. Nevertheless, if the damage in the DNA persists, p16 is triggered through p38 protein kinase-mediated mitochondrial dysfunction and ROS production. As a consequence, CDK inhibition and activation of the tumor suppressor RB1 protein occur, leading to senescence [29,62,63]. On the other hand, oxidative stress induces telomerase export from the nucleus to mitochondria, which leads to a reduction in its ability to prevent telomere shortening [64]. Furthermore, TERT has multiple telomeric and extra-telomeric functions. In the nucleus, TERT canonical activity prevents telomere shortening, but inside mitochondria, reduces superoxide production and cellular ROS levels, protecting the cells from oxidative stress and maintaining mitochondrial fitness [65,66]. When oxidative stress is severe, cell survival depends on maintaining the redox homeostasis. In stem cells, this oxidative balance is regulated by a controlled production of ROS and their scavenging through the generation of endogenous antioxidants [55]. We had previously showed that not only the production of oxygen radicals in BCPs was higher than in HVs, but also that the levels of endogenous antioxidants measured in BM plasma, such as α-tocopherol and ubiquinol-10, were decreased [22]. These findings showed that MSCs of these patients reside in a niche with high ROS levels and have a decreased capacity to neutralize them. This could explain the decrease in TERT expression and telomere shortening. In this sense, Haendeler et al. showed that treating cells with the antioxidant N-acetylcysteine (NAC) decreased the nuclear export of TERT and delayed replicative senescence [35]. Furthermore, Trachana et al. showed that MSCs overexpressing TERT had an enhanced growth potential due to their improved antioxidant capacity [26] As previously demonstrated in aged BM-MSCs, the treatment of MSCs from advanced BCPs with Ubiquinol-10 during CFU-F assays resulted in a reduction of oxidative stress and partially restored their self-renewal capacity (preliminary unpublished data) [67,68].

Furthermore, Yannarelli et al. showed, that higher telomerase expression in adult MSCs is crucial for self-renewal and osteogenic differentiation and correlates with higher expression of the stemness genes *OCT-4*, *SOX-2*, and *NANOG* [41]. The basal expression level of *OCT-4* is critical for preserving the stemness and differentiation potential of MSCs by modulating the expression of *SOX-2* and *NANOG* [69]. In this work, we found that BCPs-MSCs present a diminished expression of these genes, although we were unable to detect *NANOG* expression levels in our samples. Additionally, we had previously shown that the pool of BM-MSCs of these patients presented lower numbers of CD146^+^ cells, which exerted a decreased capacity for osteogenic differentiation. Those MSCs also expressed high levels of the major inductor of mature osteoclast, membrane RANKL and other pro-osteoclastogenic factors such as CCL-2 and MMP-9, as well as secreted less amount of the main osteoclast differentiation inhibitor decoy receptor, osteoprotegerin (OPG) [21,23,37].

In accordance with those previous findings, in this work we found increased levels of gene expression of osteoclastogenic factors like *CCL-2*, *M-CSF*, and *IL-6*. It is known that these factors regulate osteoclastogenesis and are responsible for inflammatory bone pathology and osteoporosis [70–72].

In this regard, Harkness et al. have reported that CD146 status is associated with osteogenic progenitor functions of BM-MSCs as well as with their capability for *in vivo* trans-endothelial migration and homing to injured bone sites [73]. Moreover, Gnani et al. showed that the expression of CD146 was downregulated in MSCs after prolonged *in vitro* expansion, as well as in MSCs from elderly donors in comparison to MSCs obtained from young donors [49,74].

Paradoxically, gene expression of factors associated with osteogenic differentiation, such as *RUNX-2* and *BMP-2*, was increased in MSCs from BCPs. This phenomenon can be partially explained by considering our previous results, which indicated an increased BCPs-MSCs differentiation levels towards chondrogenic lineages [21]. *RUNX-2* and *BMP-2* are factors that intervene in the osteogenic and chondrogenic differentiation cascade and both vary their expression level temporarily throughout both differentiation processes during the standard differentiation assays [75,76]. Furthermore, the expression levels of these genes were measured in cultures without external factors that induce differentiation to any of the lineages. Considering that these cells were isolated from the BM of BCPs in which pro-osteoclastogenic factors were improved [23,37], it indicates that the niche from which they come is inducing them to differentiate into osteogenic linage, regardless of whether their altered characteristics prevent them from achieving a successful osteogenic differentiation *in vitro*.

## Conclusion

Distant primary breast tumor modifies the BM-MSC pool, producing an altered phenotype. Increased oxidative stress with ROS production together with decreased TERT expression and functional ability to preserve telomere length could explain the senescent phenotype, altered gene expression profile, diminish self-renewal, and the increases in proinflammatory-proosteoclastogenic capacity observed in the MSCs of these patients. Nevertheless, functional studies are needed to confirm these findings. In the future, reversing this abnormal profile of MSCs through ROS inhibition with antioxidant therapy may be a promising approach for the treatment of these patients.

## Data Availability

All data analyzed during this study are included in this published article.
